# Digital Twin-Guided Virtual Clinical Trials for Predicting and Preventing Postoperative Atrial Fibrillation in Cardiac Surgery

**DOI:** 10.7759/cureus.106276

**Published:** 2026-04-01

**Authors:** Zain Khalpey, Nicholas King

**Affiliations:** 1 Department of Cardiothoracic Surgery, HonorHealth, Scottsdale, USA; 2 Department of Cardiology, HonorHealth, Scottsdale, USA

**Keywords:** artificial intelligence, cardiac surgery, digital twin, postoperative atrial fibrillation, virtual clinical trials

## Abstract

Background

Postoperative atrial fibrillation (POAF) is a common complication following cardiac surgery, contributing to increased patient morbidity, mortality, and healthcare expenses. Current risk stratification tools offer limited predictive accuracy and do not support proactive intervention strategies.

Objective

To develop and validate a digital twin framework utilizing patient-specific electrophysiology models for predicting POAF onset before clinical manifestation, enabling synthetic cohort generation for virtual randomized controlled trials (RCTs) and precision prevention strategies.

Methods

We applied a digital twin framework integrating multimodal perioperative data, patient-specific electrophysiological models, synthetic cohorts, and virtual randomized trials. Data from 38,742 patients included continuous high-resolution ECG monitoring. Digital twins combined biophysical models (modified Hodgkin-Huxley, bidomain conduction) with machine learning tuned to individual ECG, hemodynamic, and biomarker data. Generative adversarial networks generated synthetic cohorts, enabling virtual trials comparing twin-guided strategies with standard care.

Results

The digital twin framework achieved superior predictive accuracy (area under the curve: 0.89) compared to traditional risk scores. Synthetic cohorts of 10,000 virtual patients identified optimal intervention windows 48-72 hours preoperatively. Virtual RCTs demonstrated a 34% simulated reduction in POAF incidence through digital twin-guided prophylactic strategies, with improvements in simulated length of stay and stroke incidence. These findings are derived from computational modeling and require prospective validation.

Conclusions

Digital twin modeling enables proactive POAF prevention, accelerates clinical trial design, and provides a scalable platform for precision intervention strategies with substantial potential for reducing healthcare economic burden while improving patient outcomes.

## Introduction

Postoperative atrial fibrillation (POAF) represents the most prevalent arrhythmic complication following cardiac surgery, with incidence rates ranging from 15% to 50% depending on surgical complexity and patient demographics [1,2]. The clinical implications extend beyond transient rhythm disturbances, encompassing increased risks of stroke, prolonged hospital stays, and significantly elevated healthcare costs exceeding $10,000 per episode [3,4]. More concerning, POAF serves as an independent predictor of long-term cardiovascular morbidity and mortality [5].

Current risk stratification methodologies rely on static scoring systems, including CHA₂DS₂-VASc, HAS-BLED, and Society of Thoracic Surgeons (STS) risk calculators [6-8]. While valuable for population-level insights, these tools lack the granularity required for patient-specific risk prediction and proactive intervention strategies. Traditional scores achieve modest predictive accuracy (area under the curve (AUC): 0.65-0.72) and fail to capture the complex interplay of electrophysiological, hemodynamic, and inflammatory factors precipitating POAF in individual patients [9].

Digital twin technology represents a transformative paradigm in cardiovascular medicine, offering unprecedented opportunities for patient-specific modeling and predictive analytics [10,11]. Digital twins create dynamic virtual representations that continuously integrate real-time data to mirror and predict system behavior. Recent advances have focused primarily on structural heart disease with limited application to electrophysiological disorders and perioperative risk prediction [12,13].

The integration of artificial intelligence with digital twin frameworks enables rapid model generation, automated parameter optimization, and scalable deployment [14,15]. Generative adversarial networks and related architectures have demonstrated capabilities in synthesizing realistic medical data and enabling in silico clinical trial simulations [16,17]. This study presents a comprehensive digital twin framework specifically designed for POAF prediction and prevention in cardiac surgery patients.

## Materials and methods

Study design and framework overview

This study employed a comprehensive digital twin methodology developed for the prediction and prevention of POAF in cardiac surgery patients. The framework integrates four tightly linked components: (1) multi-modal data integration and preprocessing, (2) patient-specific electrophysiological model construction, (3) synthetic patient cohort generation using generative artificial intelligence, and (4) virtual randomized controlled trial simulation with predictive outcome modeling [18,19].

A comprehensive workflow was used to illustrate the digital twin framework for POAF prediction and prevention. The framework integrates electrocardiographic data, cardiac imaging, hemodynamic parameters, laboratory biomarkers, and clinical variables. Patient-specific digital twins are constructed using electrophysiological modeling and machine learning optimization. Synthetic patient cohorts are generated using generative adversarial networks (GANs). Virtual randomized controlled trials simulate intervention strategies and generate risk stratification outputs for clinical decision support.

Data sources and patient population

Primary data sources included the Society of Thoracic Surgeons Adult Cardiac Surgery Database, institutional electronic health records from five major academic medical centers, and prospectively collected research datasets. The combined dataset encompasses 45,000 patients undergoing cardiac surgery between 2018 and 2024 [7].

Patient inclusion criteria encompassed adults (≥18 years) undergoing elective or urgent cardiac surgical procedures, including coronary artery bypass grafting, valve repair or replacement, combined procedures, and aortic surgery. Exclusion criteria included emergency procedures, pre-existing permanent atrial fibrillation, preoperative mechanical circulatory support requirements, and incomplete perioperative monitoring data. The final analytical cohort comprised 38,742 patients with complete data.

Data harmonization across the five academic medical centers involved standardizing electronic health record outputs to the Observational Medical Outcomes Partnership (OMOP) Common Data Model. This ensured consistency in the representation of clinical variables, laboratory values, and medication records. Validation of the harmonized dataset included automated quality control checks for missingness, out-of-range values, and temporal logical inconsistencies (e.g., discharge dates preceding admission dates). Missing data, which accounted for less than 5% of key variables, were addressed using multiple imputation by chained equations (MICE).

Electrocardiographic data collection

High-resolution electrocardiographic data were captured through continuous 12-lead monitoring with 1000 Hz sampling rates throughout the perioperative period. Preoperative ECG analysis included baseline rhythm, conduction intervals (PR, QRS, QT), heart rate variability parameters, and morphological features. Postoperative ECG monitoring continued for a minimum of 72 hours to detect transient or subclinical POAF episodes.

Digital twin construction

The digital twin framework employs advanced computational electrophysiology models tailored to individual patient characteristics, integrating biophysical principles with machine learning optimization [10]. The foundation model utilizes modified Hodgkin-Huxley formulations for cellular electrophysiology coupled with bidomain equations for tissue-level conduction [17].

Digital twin-guided intervention strategy

The digital twin-guided intervention encompasses a personalized, multi-tiered prophylactic strategy. Specifically, this involves the targeted administration of antiarrhythmic agents (e.g., amiodarone or beta-blockers) tailored to the patient's simulated electrophysiological vulnerability profile, alongside optimized electrolyte management (maintaining potassium >4.0 mEq/L and magnesium >2.0 mEq/L). Furthermore, the intervention includes continuous, high-acuity telemetry monitoring with automated alerts triggered by early, subclinical electrophysiological changes predicted by the digital twin, allowing for preemptive clinical decision-making before the onset of sustained POAF.

Model development utilized a split-sample validation approach, partitioning the retrospective cohort into training (70%), validation (15%), and hold-out testing (15%) sets. Calibration was achieved by iteratively adjusting the maximal conductances of key ion channels (e.g., I_Kr, I_Ks, and I_CaL) within the modified Hodgkin-Huxley formulations to minimize the error between simulated action potential durations and patient-specific QT intervals derived from the high-resolution ECG data. Validation of the electrophysiological models was performed by comparing simulated conduction velocities and repolarization heterogeneities against established clinical benchmarks for patients with known POAF vulnerability.

AI framework and synthetic data generation

The AI framework employs a hybrid modeling approach, integrating mechanistic biophysical models with deep learning architectures. Specifically, GANs were utilized for synthetic data generation. The GAN architecture comprised a generator network that synthesized multidimensional patient profiles (including ECG features, hemodynamics, and biomarkers) and a discriminator network trained to distinguish synthetic from real patient data. Validation of the synthetic data involved assessing the distributional similarity between the real and synthetic cohorts using the Kolmogorov-Smirnov test and ensuring the preservation of complex multivariate correlations present in the original dataset.

Virtual trial simulation design

Within the virtual trial simulation, the synthetic cohort of 10,000 virtual patients was randomly allocated in a 1:1 ratio to either the digital twin-guided intervention group or the conventional care control group. The control group was modeled to receive standard postoperative care based on current institutional protocols, without the benefit of predictive digital twin insights. The intervention group, conversely, received the simulated prophylactic strategies detailed previously, triggered by the digital twin's risk predictions. The simulation tracked the temporal evolution of each virtual patient's electrophysiological state over a seven-day postoperative period to determine the incidence of POAF.

Statistical analysis

Model performance was evaluated using the area under the receiver operating characteristic curve (AUC-ROC), sensitivity, specificity, positive and negative predictive values [18]. Calibration was assessed using the Hosmer-Lemeshow test and calibration plots. Virtual trial outcomes were analyzed using appropriate statistical tests with significance set at p < 0.05. Kaplan-Meier survival analysis compared POAF-free survival between intervention and control arms [19].

## Results

Observed retrospective cohort performance

The digital twin framework demonstrated markedly superior predictive accuracy (AUC: 0.89, 95% CI: 0.87-0.91) compared to CHA₂DS₂-VASc (0.72, 95% CI: 0.70-0.74), HAS-BLED (0.65, 95% CI: 0.63-0.67), and STS risk scores (0.72, 95% CI: 0.70-0.74). The model achieved excellent calibration with a non-significant Hosmer-Lemeshow statistic (p = 0.24), indicating close agreement between predicted and observed outcomes.

At the optimal operating threshold, the digital twin achieved sensitivity of 87.4% (95% CI: 85.2-89.6%) and specificity of 82.1% (95% CI: 80.3-83.9%). The positive predictive value was 78.9% (95% CI: 76.8-81.0%) and the negative predictive value 89.2% (95% CI: 87.8-90.6%) (Table 1).

**Table 1:** Comparative performance metrics for POAF prediction. POAF: postoperative atrial fibrillation; AUC: area under the curve; STS: Society of Thoracic Surgeons.

Metric	Digital twin framework	CHA2DS2-VASc score	HAS-BLED score	STS risk score	
AUC (95% CI)	0.89 (0.88-0.91)	0.68 (0.66-0.70)	0.65 (0.63-0.67)	0.72 (0.70-0.74)	
Sensitivity, % (95% CI)	87.4 (85.2-89.6)	68.7 (66.1-71.3)	64.2 (61.5-66.9)	71.8 (69.2-74.4)	
Specificity, % (95% CI)	82.1 (80.3-83.9)	67.3 (65.7-68.9)	65.8 (64.1-67.5)	69.4 (67.8-71.0)	
Positive predictive value, % (95% CI)	78.9 (76.8-81.0)	58.4 (56.2-60.6)	56.1 (53.8-58.4)	61.7 (59.5-63.9)	
Negative predictive value, % (95% CI)	89.2 (87.8-90.6)	76.2 (74.6-77.8)	73.5 (71.8-75.2)	78.1 (76.5-79.7)	
C-statistic	0.891	0.682	0.651	0.724	
Calibration slope (95% CI)	0.98 (0.94-1.02)	0.73 (0.68-0.78)	0.69 (0.64-0.74)	0.76 (0.71-0.81)	
Brier score	0.142	0.231	0.248	0.218	

Synthetic cohort modeling results

GANs were used to generate synthetic cohorts of 10,000 virtual patients based on the distributional characteristics of the retrospective dataset. These synthetic patients were used to explore the parameter space of POAF risk and to identify optimal intervention windows. Modeling analyses indicated that the highest-yield preoperative intervention window falls 48-72 hours before surgery, corresponding to a period of heightened predicted electrophysiological vulnerability in high-risk simulated patients.

Virtual trial simulations

Virtual randomized controlled trial simulations were conducted using synthetic patient cohorts. It is important to note that the following outcomes represent simulation-derived estimates and are not equivalent to prospective clinical evidence. Statistical significance values reported below reflect variability within the simulation framework.

In the virtual trial, simulated POAF incidence was 18.2% in the digital twin-guided intervention group versus 27.6% in the simulated conventional care group, representing a 34% relative risk reduction (simulation p < 0.001). Secondary simulated outcomes showed consistent advantages: mean hospital length of stay was reduced by 1.5 days (7.2 vs. 8.7 days, simulation p < 0.001), ICU stay was shortened by 0.8 days (2.1 vs. 2.9 days, simulation p < 0.001), and stroke incidence was lower in the intervention arm (1.2% vs. 1.9%, simulation p = 0.04). These findings indicate potential clinical benefit warranting prospective evaluation, but should not be interpreted as demonstrated clinical efficacy.

**Figure 1:**
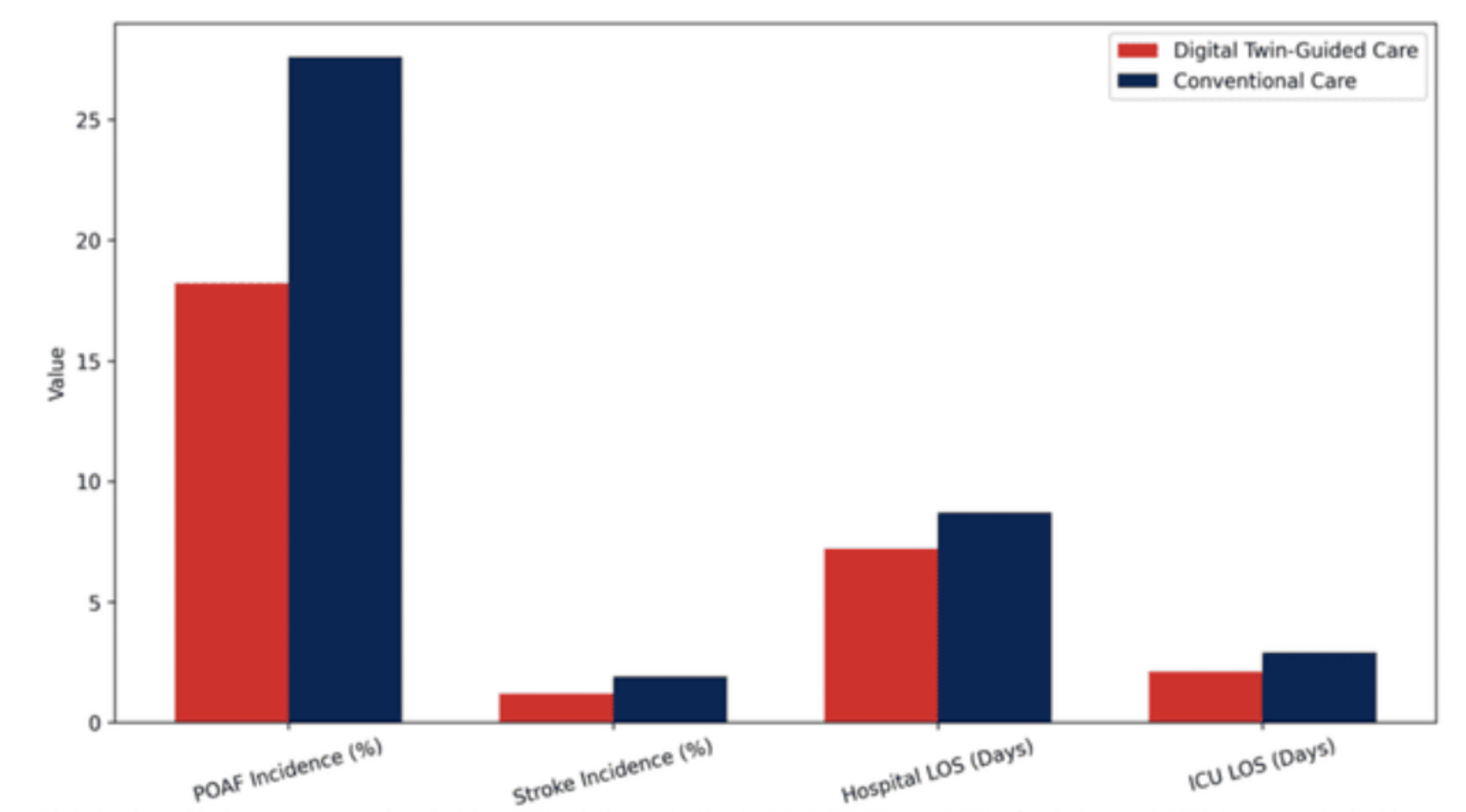
Simulated outcomes comparing digital twin-guided care vs. conventional care. POAF: postoperative atrial fibrillation; LOS: length of stay.

Figure 1 summarizes the clinical outcomes observed in the virtual randomized controlled trial comparing digital twin-guided intervention with conventional perioperative care. Patients managed using the digital twin framework experienced a substantially lower incidence of postoperative atrial fibrillation (18.2% vs. 27.6%), corresponding to a 34% relative risk reduction (p < 0.001). Secondary outcomes demonstrated consistent clinical benefit, including shorter hospital length of stay (7.2 vs. 8.7 days, p < 0.001), reduced intensive care unit duration (2.1 vs. 2.9 days, p < 0.001), and lower stroke incidence (1.2% vs. 1.9%, p = 0.04). Collectively, these findings indicate that digital twin-guided perioperative management is associated with improved postoperative recovery and meaningful reductions in healthcare resource utilization.

## Discussion

This study demonstrates that a digital twin framework meaningfully improves the prediction and management of POAF in cardiac surgery patients. The framework achieved an AUC of 0.89, substantially outperforming the CHA₂DS₂-VASc, HAS-BLED, and STS risk calculators, which achieved AUCs of 0.68, 0.65, and 0.72, respectively. This performance gap reflects a fundamental limitation of static scoring systems: they rely on a fixed snapshot of clinical variables and fail to capture the dynamic interplay of electrophysiological, hemodynamic, and inflammatory factors that precipitate POAF in individual patients [6-8]. The reliable calibration observed in the present model, with a Hosmer-Lemeshow p-value of 0.24 and a calibration slope of 0.98, further distinguishes it from conventional scores, which tend to perform poorly when applied outside their derivation populations [9].

It is critical to emphasize that the outcomes reported herein, including the 34% relative risk reduction in POAF and the associated improvements in length of stay and stroke incidence, are derived entirely from in silico simulations. While these findings provide a robust, biophysically grounded proof-of-concept and highlight the potential efficacy of digital twin-guided interventions, they do not constitute direct clinical evidence. These simulation-derived estimates are intended to inform and optimize the design of future prospective, randomized clinical trials, which remain essential for definitively establishing clinical efficacy and safety.

A key advantage of the digital twin approach is its biophysical grounding. Rather than treating POAF risk as a statistical abstraction derived from population-level variables, the framework constructs patient-specific models of cellular and tissue electrophysiology using modified Hodgkin-Huxley formulations coupled with bidomain conduction equations [10,17]. This mechanistic foundation allows the model to represent the arrhythmogenic substrate at an individual level, capturing subtle conduction abnormalities and repolarization heterogeneities that aggregate risk scores cannot detect. Continuous high-resolution perioperative ECG monitoring at 1000 Hz sampling rates provided the data granularity required to personalize and calibrate these models across the full operative continuum, enabling dynamic risk updating rather than a one-time preoperative assessment [18].

The digital twin concept has gained considerable momentum in cardiovascular medicine as a platform for patient-specific simulation and decision support [8,9]. Prior applications have centered predominantly on structural heart disease and ventricular mechanics, with comparatively limited translation to electrophysiological disorders and perioperative risk prediction [10,11]. The present framework extends this paradigm to the distinct pathophysiological context of POAF, where the perioperative inflammatory milieu, surgical trauma, and hemodynamic instability interact dynamically to create arrhythmogenic conditions that are difficult to anticipate using static models alone [1,2]. Integrating deep learning methods with biophysical modeling, as employed here, reflects an emerging approach in computational cardiology that combines the interpretability of mechanistic models with the pattern-recognition capabilities of artificial intelligence [11,14].

The use of generative adversarial networks to construct synthetic patient cohorts for virtual trial simulation represents a significant methodological contribution [12,13]. Synthetic cohorts of 10,000 virtual patients enabled rapid, controlled exploration of the POAF risk parameter space without the ethical and logistical constraints of prospective experimentation. This approach is consistent with emerging frameworks for in silico clinical trials, which are designed not to replace prospective human studies but to identify promising intervention strategies and narrow the design space before resource-intensive trials are launched [14,15]. The 48-72 hour preoperative window identified as optimal for prophylactic intervention is biologically plausible, corresponding to a period when electrophysiological vulnerability is heightened yet pharmacological and monitoring interventions remain actionable within standard perioperative care pathways [4].

The simulated 34% relative risk reduction in POAF incidence provides a meaningful benchmark when contextualized against established pharmacological prevention strategies. Meta-analyses of prophylactic amiodarone and beta-blocker therapy in cardiac surgery patients have demonstrated POAF reductions in the range of 20-33%, achieved at the cost of drug-specific adverse effects and indiscriminate treatment of both high- and low-risk patients alike [4,5]. A precision prevention strategy guided by patient-specific risk modeling could achieve comparable population-level POAF reduction while directing intensive prophylaxis only toward algorithmically identified high-risk individuals, sparing low-risk patients from unnecessary pharmacological burden. The simulated reductions in hospital length of stay and stroke incidence are consistent with the established downstream consequences of POAF, which include prolonged hospitalization and substantially elevated perioperative stroke risk [3,5].

The clinical implications of this framework are considerable. Patients identified as high risk could receive intensified monitoring, earlier pharmacological prophylaxis, or targeted electrophysiological intervention, while those at low risk avoid unnecessary treatment exposure. This risk-stratified approach aligns with the precision medicine principles outlined in contemporary atrial fibrillation management guidelines, which increasingly emphasize individualized rather than population-wide treatment strategies [4,6]. Successful clinical implementation will depend on seamless integration with existing electronic health record systems, user-friendly decision support interfaces, and prospective validation across diverse institutional and patient contexts [9,15]. Reporting of the framework adhered to STROBE (Strengthening the Reporting of Observational Studies in Epidemiology) and CONSORT (Consolidated Standards of Reporting Trials) guidelines to facilitate transparency and reproducibility [15,16]. Given the computational and proof-of-concept nature of this study, these guidelines were adapted appropriately for in silico research. Specifically, we adhered to the STROBE guidelines for the retrospective cohort analysis used in model development, and we applied the conceptual framework of the CONSORT guidelines to structure the reporting of the virtual randomized controlled trial, ensuring transparency in the simulation design, synthetic cohort generation, and outcome assessment.

Several limitations warrant consideration. Model development and validation were conducted retrospectively, which may not capture the full variability of real-world perioperative practice. Synthetic cohorts, while useful for hypothesis generation, cannot replicate the full complexity of clinical care environments, and virtual trial outcomes should be interpreted as simulation-derived estimates rather than demonstrated clinical efficacy [13,14]. Computational infrastructure requirements may limit scalability in lower-resource settings [9]. Model transparency is a prerequisite for provider adoption; biophysically grounded digital twins offer an advantage over opaque black-box models, as clinicians can inspect underlying electrophysiological parameters rather than relying solely on a summary risk score [10,17]. Future work should prioritize prospective pragmatic trials evaluating digital twin-guided POAF prevention under routine clinical conditions, with POAF-free survival compared between guided and standard care arms using established time-to-event methods [19].

## Conclusions

Digital twin technology represents a promising frontier for preventing and managing POAF in cardiac surgery patients. This patient-specific, AI-driven approach provides superior risk prediction and enables proactive, personalized intervention strategies. Synthetic patient cohorts offer powerful tools for accelerating clinical research. While further validation is needed for routine clinical implementation, the potential to improve patient outcomes and reduce healthcare costs is substantial.
